# What’s so special about empirical adequacy?

**DOI:** 10.1007/s13194-017-0171-7

**Published:** 2017-03-07

**Authors:** Sindhuja Bhakthavatsalam, Nancy Cartwright

**Affiliations:** 1 Liberal Studies Program, California State University, Northridge, Northridge, CA USA; 2 Department of Philosophy, University of California, San Diego, La Jolla, CA USA; 3 Department of Philosophy, Durham University, Durham, UK

**Keywords:** Scientific realism, Antirealism, Scientific understanding, Pragmatism, Aims of science, Empirical adequacy, Scientific truth

## Abstract

Empirical adequacy matters directly - as it does for antirealists - if we aim to get all or most of the observable facts right, or indirectly - as it does for realists - as a symptom that the claims we make about the theoretical facts are right. But why should getting the facts - either theoretical or empirical - right be required of an acceptable theory? Here we endorse two other jobs that good theories are expected to do: helping us with a) understanding and b) managing the world. Both are of equal, often greater, importance than getting a swathe of facts right, and empirical adequacy fares badly in both. It is not needed for doing these jobs and in many cases it gets in the way of doing them efficiently.

## Our thesis

*Theory choice* has long been a prime topic in philosophy of science: ‘How should we choose from among competing theories?’ Theory virtues have also in recent years become a standard, closely related topic: “What virtues should a desirable theory have?” “Are the preferred candidates (perhaps simplicity, heterogeneity, or explanatory power) symptomatic that a theory is true?” We think that most of this discussion is misguided. The fundamental problem is that, even where truth has been eschewed as the aim in view, the considerations raised for theory choice are still trained on truth – and on its fellow traveler, empirical adequacy, the sine qua non for truth. Even if we want other virtues in a theory like say, simplicity, it is assumed that we first winnow down to theories that are (at least roughly) empirically adequate. We think that this approach is off on the wrong foot: empirical adequacy is a poor starting point that could have us picking from among the wrong theories in many contexts. For many purposes that science aims to serve, the theories we choose need not be anything like empirically adequate, and as we will argue, in some cases empirical adequacy is a positive vice.

## Empirical adequacy and theoretical virtues

It is good to start with a definition of “empirical adequacy”, but it turns out to be difficult to find one in the philosophical literature.^1^ We think that what is usually intended is something like this: a theory (or model or set of scientific claims) is empirically adequate when the claims it makes about empirical phenomena – or at least the bulk of these claims, or the central ones – are correct, or approximately correct enough, where some distinction between empirical and theoretical phenomena is supposed. We shall assume this usage and leave aside the question of what counts as empirical and what as theoretical. The question at the heart of this paper is, ‘Why privilege empirical adequacy among theoretical virtues?’

The mistake about requiring empirical adequacy of our theories is connected with a familiar but ill-explained notion associated with theory choice – that of *acceptability*. It is often debated what makes a theory acceptable. But whatever it is, empirical adequacy is almost always taken as a basic requirement for a theory to be acceptable. But what *is* acceptability? Many philosophers associate acceptability with practical ends, but not without also associating it with knowledge, belief, or other epistemic concepts. For instance according to Ernan McMullin (1982),

“When a physicist “accepts” a particular theory, this can mean that he believes it to be the best-supported of the alternatives available or that he sees it as offering the most fruitful research-program for the immediate future. These are *epistemic* assessments; they attach no values to the theoretical alternatives other than those of likelihood or probable fertility. On the other hand, if theory is being applied to practical ends, and the theoretical alternatives carry with them outcomes of different value to the agents concerned, we have the typical decision-theoretic grid involving not only likelihood estimates but also “utilities” of one sort or another.”(p.8).

For McMullin, in theoretical science acceptance is purely epistemic. Where theory is applied to practical ends, even there acceptance involves epistemic assessments like “likelihood estimates”, although other practical values may be factored in as well. At any rate, we have no quarrel with empirical adequacy as an indicator of theory acceptability when acceptability is to be judged in terms of truth: a theory with false implications cannot be true, whether its implications are about empirical phenomena or something else. But there are lots of other things one can intend by labeling a theory ‘acceptable’.

According to Richard Rudner (1953) for instance, to accept is to “approve as a basis for a specific kind of action.” (p.2) And according to Jonathan L. Cohen (1995) “to accept that *p* is to have or adopt a policy of deeming, positing, or postulating that *p*—i.e. of including that proposition or rule among one’s premises for deciding what to do or think in a particular context, whether or not one feels that it is true that *p*.”(p.4) It is these senses of accept that we engage with here. “Acceptable” in these senses is (at least) a 2-place relation: things are not just accepted full stop, but rather *for some purposes* and not for others. Scientific theories are employed for a great variety of purposes. Empirical adequacy is necessary for none we can think of besides truth of the theory. (Of course, many non-realists who are not concerned with truth are also preoccupied with empirical adequacy. Bas van Fraassen’s *The Scientific Image* is a classic example. The aim of science, says *The Scientific Image*, is to produce theories that are empirically adequate – even though truth is not the ultimate aim. We return to this below).

Put baldy, our thesis may seem obvious. But it does not seem to be so in practice. Consider the discourse on theoretical virtues. At least since Popper (1935) and Kuhn (1977) these have almost always been presented as characteristics one might wish for in a theory *in addition to empirical adequacy.* For instance, according to Heather Douglas (2013), “There are values that are genuinely truth assuring, in the minimal sense that their absence indicates a clear epistemic problem.” (p. 799) These according to her are the basic, minimal criteria for adequate science, and they include empirical adequacy and internal consistency; the others are mere “ideal desiderata”. Douglas admits that in practice scientists may aim for characteristics like simplicity and potential explanatory power even when the minimal criteria of empirical adequacy and internal consistency fail. But, she maintains,

This must be done with the full acknowledgement that the theory is inadequate as it stands, and that it must be corrected to meet the minimum requirements as quickly as possible. Although philosophers like to quip that every scientific theory is ‘born falsified’, no scientist should be happy about it. (p. 802)

However, she concedes that in addition to the minimal criterion of empirical adequacy (and internal consistency) – which, as she describes, is a virtue of a theory “in relation to evidence” – it would be good to aim for other virtues, like simplicity and scope.

When truth is the goal, it makes sense to take empirical adequacy as a requirement and other virtues as desiderata, useful for other purposes in addition. But there is an opposing standpoint that does not take truth to be the goal of science, maybe because there is no such thing as *the* goal of science, or because truth is unobtainable, or because we would never know it we had it, or because one wants science to do something other than accumulate truths, possibly many other things. Douglas is right in that lack of empirical adequacy poses an *epistemic* concern. But science often has concerns that are not epistemic. In this context our question is not just a matter for philosophy. It has serious cash value. If we need a theory to do a job that can be done well even by theories that are false or empirically inadequate, why restrict our choice to those that are empirically adequate? Consider an analogy. We need a knife to cut bread. Why buy a multitasking Swiss Army knife that is not only more expensive than a bread knife but also cuts bread considerably less well? Similarly, when we want a theory that’s simple but need not be true to do a job in view, why take on the dual burden of finding a theory that is both empirically adequate and simple? Surely this is foolish unless we have reason to think empirically adequate theories serve the purposes at hand – those that simplicity is supposed to serve – better than theories that are not empirically adequate. As with knives for cutting bread, we shall argue, so too with theories. Demanding a virtue that fits the theory for an additional job can not only be costly but it can narrow our choices to ones that don’t do the job we want at all well.

So far we have been discussing the pursuit of empirical adequacy as a stepping-stone to finding a true theory. But well-known anti-realists have also embraced empirical adequacy, for instance the logical positivists. Why? How did they answer our question: what’s so special about empirical adequacy? Many supposed that only claims that can be confirmed by observation are meaningful. But under the influence of Hanson, Feyerabend, Kuhn, and others, most philosophers have come to reject i) the theory of meaning that this claim presupposes, ii) the possibility of distinguishing what can be confirmed through observation and what not (do we see through a microscope?), and iii) the theory neutrality of observation itself.

Van Fraassen’s *The Scientific Image* (1980) champions an empiricism that avoids these pitfalls; what he calls “constructive empiricism”: the aim of science is to be empirically adequate, where, as he defines it, a theory is empirically adequate if it gets right the facts it implies about the objects that are observable by us. He avoids the pitfalls by arguing that it is a matter of empirical fact what objects are and are not observable by us, by contrast with the question of which features of objects are theoretical and which observable. He tells us, in addition, that while accepting a theory has to do with pragmatics and with how well the theory serves specific purposes*,* like McMullin, he takes it that *to accept a theory is also to take the theory to be empirically adequate*: this does not sound like a definition but rather an injunction for him.

With respect to acceptance, as we said above, our focus is on receiving the theory for specific purposes, and this does not seem to be shared by van Fraassen given his discussion of theory virtues. He allows that scientists may in pursuit of usable and useful theories demand pragmatic virtues that may have nothing to do with saying things as they are about the empirical world, like simplicity, scope, and explanatory power. He also does not argue for the superiority of empirical adequacy over pragmatic virtues in settling on what theory to use or to pursue. This may make it sound as if our views converge. But no. According to *The Scientific Image* while scien*tists* can have pragmatic aims, the aim of *science* is empirical adequacy. This simply invites a reformulation of our question: what’s so special about this aim from among all others? Van Fraassen does not give good reasons for why this should be so.

Van Fraassen does distinguish empirical adequacy as a ‘semantic’ virtue – one having to do with saying things about the world the way they are – as opposed to the other ‘pragmatic’ virtues. But if we are not aiming for true theories, then why care especially for semantic virtues? If we’re not aiming for true theories, the fact that empirical adequacy is a necessary condition for success at that aim becomes irrelevant and there must be some other reason to aim for empirical adequacy. We can think of one, which we introduce by another analogy. We do not think that this is the kind of defense that either van Fraassen or the realists would choose to mount. But then we pose this as a challenge for further discussion: if not this, then what?

Suppose your sole concern is shopping and you want to be assured of a certain level of reliable quality without your having to do any thinking at all, regardless of whether you are buying pickles or sunscreen, a sweater or a refrigerator. And this demand for quality coupled with a demand for a one-stop-shop for everything with minimal thought and research trumps all. Then you should be willing to drive hundreds of miles, spend vast amounts, and stand in long check-out queues to get access to a shop or set of shops that sells everything you need and of the quality you want. You should be willing to do so even if a variety of local shops can supply the very goods you buy, of the same quality and far more cheaply – but you would have to think about which shop to go to for which goods (and not, for instance, expect the butcher to sell you a sweater), and you would have to notice that some of the local shops have shoddy offerings. Similarly imagine your sole concern is to predict a variety of empirical facts accurately; you have no other aim. You couple that with the demand that you should be able to do so without ever having to think what theory to use for what prediction. Then aiming for a large set of empirically adequate theories makes good sense: we would be able to use any theory from within that set without thought to make empirical predictions. But it can be excessively costly and it can get in the way of important aims you might have other than predicting empirical results without having to reflect on the theory to use to do so.

To make this case, we will focus on two very broad purposes of science and scientists shared across many scientific endeavors: a) gaining understanding of the world and b) managing the world. We choose these two because they are widely accepted as fundamental goals of science and because these are two areas where we have found others very resistant to our campaign against empirical adequacy. Sure, ‘getting it right’ about *something or other* can matter to these two jobs, but getting these things right would not count as empirical adequacy in anybody’s books. Theories can do these two jobs very well and still be very wrong in the bulk of their empirical predictions. Our view is similar to Elliot and McKaughan’s (2014):

Scientists need not always maximize the fit between a model and the world; rather, the purposes of the users determine what sort of fit with the world (and therefore what balance between epistemic and non-epistemic considerations) is needed in particular contexts. Scientists use models and theories to represent the world for specific purposes, and if they can serve those purposes best by sacrificing some epistemic features for the sake of non-epistemic ones, it is entirely legitimate for them to do so. (4)

We concede that truth – or for many anti-realists, just plain empirical adequacy – might be important for various reasons: for its own sake, for finding some kind of teleological meaning or purpose to one’s scientific life and work (taking one’s theories to be heading towards the truth might give meaning to a scientist’s work), etc. But to *mandate* empirical adequacy as a minimum criterion for a scientific theory is entirely unreasonable and just wrong.

One consequence of dislodging empirical adequacy from pride of place in theory choice that we hope will follow in train is a new set of focuses in the discourse on theoretical virtues. Virtues must be fit for purpose. If different theories are chosen on different occasions to serve different purposes, we almost certainly will find on closer inspection that scientists call a great number of virtues into play beyond philosophy’s usual suspects. What are they? Which virtues are used in practice to select for which purposes? Can we find warrant for these practices?

Although we provide several examples from scientific practice to illustrate our arguments, we want to urge that these are illustrations, not arguments. Our normative account of the role of empirical adequacy in science does not depend on the (descriptive) examples from scientific practice. Our arguments against the primacy of empirical adequacy among theoretical virtues stand on their own and should be persuasive – we hope – even without theses instances from scientific practice to illustrate and support them.

## In pursuit of understanding

Here we don’t give a single account of understanding, and for a good philosophic reason. Understanding, we claim, is not a natural kind; it is an ordinary human concept. It is used almost everywhere, from everyday life to the natural and social sciences, history, theology, the law courts, and the arts, in different loosely related ways to serve different loosely related purposes. We will look instead at many currently popular accounts in philosophy of science, which we group into three categories, that arguably capture reasonable, though different, senses of scientific understanding, to see how empirical adequacy fares with them.

Scientific understanding, as Regt et al. (2009) have pointed out, is a three-term relation involving a model, explanation, or other vehicle, the target that we want to understand, and an agent, the understander. Although agency is an important aspect of scientific understanding, there seems to be a rough division of labor in philosophy. Epistemology focuses on agency – what characteristics an agent must have and what they must do in order to understand (cf. the recent spate of work in epistemology on ‘grasping’^2^). Philosophy of science has by contrast not been much concerned with what is in the heads of agents, or what they do in understanding something, but rather with the public products of science that can provide understanding, the *vehicles* of understanding – explanations, theories, models etc. That is what our discussion will focus on.

One could categorize types of understanding discussed in recent philosophy of science literature according to kinds of vehicles (e.g theories, models, narratives, and images) or perhaps according to what is to be understood (e.g. a happening, a general phenomenon, a regularity, a domain of happenings, even “the world as a whole”). To explore our claim, we find an alternative categorization – realist, counterfactual, and pragmatic understandings – more useful. As you will notice these classes are only roughly characterized; nor are they mutually independent since there are examples that fall into multiple categories. But they should suffice to show that empirical adequacy is not needed for understanding as understanding is conceived on a great many current philosophic accounts. We shall spend the most time on realist understanding because we take it that this is where the case for empirical adequacy is strongest. We shall then turn to other senses of ‘scientific understanding’ that philosophers have developed in which the importance of empirical adequacy does not seem so apparent from the start.

### Realist understanding

Realist, yes, but realist about what? In the philosophy of science literature ‘realist’ usually has to do with whatever it is that theory presents as responsible for, or (in whatever is one’s preferred sense) ‘explaining’ phenomena, such as ‘underlying’ causes, structures, general principles, or theoretical laws–. We shall start there, considering cases where the understanding of some phenomena is provided by a theoretical representation of the laws or causes supposed to be responsible for them, and we shall suppose, since we are dealing here with ‘realist’ understanding, that understanding requires that the representation get it right, or at least right enough about what matters most.

Besides theoretical laws and underlying causes, one can be realist about a good many other things. For instance, suppose one has what might be thought of as a thoroughly empiricist notion of understanding, that for a theory or model to provide understanding, it has to get right the empirical facts that follow from it. Here one is being ‘realist’ about the empirical facts. This kind of realist understanding would of course require empirical adequacy. Many other accounts of understanding look to something that is neither theoretical laws and causes nor just empirical facts. They look to things like the ‘overall picture’, the ‘world as a whole’, the ‘patterns’, the ‘similarities’, or the ‘categories’ and ‘natural kinds’ in nature. One can be realist about these too: there is indeed a forest to be seen, not just the trees; there genuinely are patterns; things genuinely are similar or dissimilar in ways that matter; some category schemes represent *natural* kinds, not just classifications we impose on the world. *Realist* understanding in these cases means getting the right overall picture, or representing patterns, similarities or categories as they really are.

We should note that here we neither endorse nor deny realism about any of these. Each has been linked with understanding, and in each case it seems the understanding could be either realist or pragmatist. (We get to the latter at the end of this section.) We take the realist interpretation because it poses the bigger challenge to our views. After all, it seems far less surprising that an erroneous representation of laws or causes or patterns will produce erroneous empirical predictions than that a true one will.

#### Understanding via vehicles that get the theory approximately right

Here is one widely held view about scientific theories and theoretical models: in order for a theoretical account to give us genuine understanding of a phenomenon, the theory has to get (at least most of) the theoretical facts cited in the account right. Truth about theoretical causes and laws may not be sufficient for understanding – we might demand a theory or model to be visualizable, simple, explanatory, etc., but – it’s thought – it is necessary.^3^ We do not agree with this thesis since we embrace the variety of kinds of understanding that we discuss. However, we think this is *a* significant kind of understanding that many scientists aim for, and they often intend it to be *realist* understanding. Our point here is that even when the understanding of a phenomenon is via seeing the theoretical laws or causes responsible for it and even when what we’re aiming for is a kind of realist understanding, the vehicle that provides that understanding need not be empirically adequate.

How is this possible? The short answer is that understanding comes in degrees. A vehicle can be empirically inadequate – hence theoretically not ‘all true’ – but can get right some or many of the important theoretical features of the target and hence afford a *degree* of (realist) understanding of it.

It may be useful to think in terms of two different kinds of case here. One is the familiar case of ‘idealizing models’, on which there is a lot of literature. Roughly, these get some of the significant theoretical structure of what is to be understood fairly precisely right. The second, which we call ‘rough proximates’, gets significant parts, or perhaps all, of the structure right but only very roughly. In this case, we may think of the vehicle as providing understanding because it stands in stark contrast with what is otherwise available, which is not even roughly right. Sometimes, perhaps because for the purposes for which one wants to understand something, the departure in detail from the right account does not matter. We suspect there is far more to be said about these and we try to separate them from the ‘idealization’ cases to encourage more attention to them. We’ll discuss these first, then turn to ‘idealizations’.

##### Rough proximates

Take the Rutherford model of the atom. Today the model is considered to be theoretically grossly inaccurate. According to modern quantum mechanical models, the electron does not revolve around the nucleus in planetary orbits as the Rutherford model pictures. The model is also empirically inadequate: it predicts that the electron will continuously lose energy and spiral into the nucleus causing the atom to collapse – and of course, atoms don’t collapse for if they did, matter wouldn’t exist the way it does. Despite these flaws the Rutherford model affords us some degree of (realist) understanding of atomic structure.

As realist understanding would have it, suppose we take correctness of theoretical features as one standard for evaluating the understanding provided by a model and take our current models to be more correct than older ones. Then the Rutherford model was on the path to these more correct models – it was significantly more correct than its predecessor, the plum pudding model, which takes the atom to be a ‘pudding’ of positive charge with electrons embedded in it. The Rutherford model was part of a chain of continually improving models comprising the Bohr model, the Bohr-Sommerfeld model, and the modern cloud model. As Catherine Elgin (2009) points out, understanding comes in degrees. We can think of the Rutherford model as a starting point. After all, it tells us that the positive charges in an atom are concentrated in a central nucleus containing protons and neutrons, and electrons surround it – a feature it shares with even the most modern model of the atom.

There is one clear sense of understanding – the sense of realist understanding – in which a model that gets just right the theoretical features of a target phenomenon and is hence empirically adequate can be taken to give us great understanding. In the same sense, one that is false and empirically inadequate can give us *some, less-than-perfect* (realist) understanding of the target owing to being somewhere in the vicinity of the true theoretical story: the atom is *something like* what the model says, and the model is better than others that are nowhere close to the theoretically true story. But why settle for partial understanding? For one, it is better than no understanding at all, and further, there are many situations in which our aim is ‘some understanding’ – when explaining things to children for instance, where the correct/true story can be too complex. For example, as Elgin (2009) points out, a child’s understanding of evolution according to which humans descended from apes is better than one according to which humans descended from butterflies. This could be a reason why the Rutherford model still finds a place in school science textbooks.

So models and theories that depart from the full theoretical truth and that may in consequence make wrong predictions about significant empirical facts^4^ can still provide partial realist understanding. So empirical adequacy is not necessary for partial realist understanding. More, it is not even a good clue. It might be presumed that ceteris paribus, if *V*_*1*_ and *V*_*2*_ are both vehicles of understanding of a phenomenon *X*, and *V*_*1*_ is more empirically adequate than *V*_*2*_, *V*_*1*_ provides better partial realist understanding of *X* than *V*_*2*_.^5^ This would be a mistake. There are a great many models that get right a great number of central empirical predictions, including ones that are deemed central from the point of view of a ‘true’ account, and yet are wide off the mark theoretically. This brings us to into the very familiar, much worked over philosophical territory of underdetermination, unconceived alternatives, and the like, so we will not have more to say here.

##### Idealizations

Scientific models often contain idealizations, exaggerations, and omissions of certain features of the target and thus deviate from the true theoretical story, and in consequence can be empirically inadequate. How do idealized models give understanding? There is a large literature on models; since we are concerned narrowly with understanding here, we concentrate on Elgin, who addresses this explicitly. Elgin (2012) calls idealizations, omissions etc. that enhance understanding, ‘felicitous falsehoods’. According to Elgin^6^ an idealized model can exemplify – highlight, exhibit, or display – characteristics it shares with the true causes, laws, or mechanisms responsible for the phenomenon it purports to explain. In doing so, Elgin argues, the model provides understanding of, and affords epistemic access to, those features in a way a more accurate model would not because the more accurate model introduces complexities that mask the features we care about. So it can provide more understanding than one that is more accurate but more complicated.

Idealized models of the kind Elgin discusses are likely to be empirically inadequate owing to the several theoretical falsehoods they contain. One nice example comes from Nobel Prize winning economist Rodolfo Manuelli (1986), commenting on the models of another Chicago School Nobel Prize winner, Edward Prescott:

.... consider the models Prescott surveys ... Most of them are representative agent models. Formally, the models assume a large number of consumers, but they are specialised by assuming also that the consumers are identical. One of the consequences of this specialisation is a very sharp prediction about the volume of trade: it is zero. If explaining observations on the volume of trade is considered essential to an analysis, this prediction is enough to dismiss such models. But if accounting for individual fluctuations beyond the component explained by aggregate fluctuations is not considered essential to understand the effects of business cycles, the abstraction is not unreasonable. A case can even be made that if what matters, in terms of utility, is the behavior of aggregate consumption and leisure, then any model that helps explain movements in the two variables is useful in evaluating alternative policies. This usefulness is independent of the ability of the model to explain other observations. (5)

In this model – as Elgin would (rightly) claim – the effects of the aggregate behavior would be obscured if we took into account individual fluctuations. We gain understanding since the model depicts vividly what is supposed to be the correct mechanism for generating a business *cycle*, which depends on average behavior, though at the cost of getting woefully wrong some effects that depend on the distribution.

One specific kind of idealization that illustrates our point is what Cartwright (2006) calls “Galilean thought experiments” and Uskali Mäki (1994), ‘isolating models’: models that study what a single one (or small set) of the many causes of an empirical effect in a target setting contributes separately. This kind of vehicle necessarily distorts the setting in which the effect occurs and the effect it predicts will be different, often dramatically, from the effect that happens. But they nonetheless get right just what the particular cause in question contributes to that overall effects. They provide genuine realist understanding of an element of the theoretical structure responsible for that effect and how that element contributes.

#### Understanding via vehicles that get other things that matter right

Here we take up some oft-discussed aspects of ‘unification’ in the philosophy of science literature.

##### The “overall” picture

Many philosophers of science have urged unification as a source of understanding. Michael Friedman (1974) is one famous example. According to Friedman, the understanding unification provides is global as opposed to local. Unifying explanations may not increase our understanding of independent phenomena, but they increase our understanding of phenomena overall. They do so by giving us a picture of the world ‘as a whole’ not just as a collection of separate parts. He explains: “From the fact that *all* bodies obey the laws of mechanics it follows that the planets behave as they do, falling bodies behave as they do, and gases behave as they do. …. [W]e have reduced a multiplicity of unexplained, independent phenomena to one”. (15, italics as in original) For Friedman this reduction is the very “essence of scientific explanation”: “A world with fewer independent phenomena is, other things equal, more comprehensible than one with more.” (15) As Friedman pictures it, this kind of understanding by unification requires that the unifying theory be true. It is supposed that it is a *fact* that all bodies obey the laws of mechanics, a fact that embraces a good many others. In consequence the unifying theory must also be empirically adequate.

But a unifying theory need not state the facts to give us a true picture – the *right* picture – of the world (or of a particular domain within it) ‘as a whole’, reducing the number of independent phenomena and making the world more comprehensible. We can invoke Cartwright’s (1980) early arguments from *The Truth Doesn’t Explain Much* 7 about what are generally deemed to be our very best unifying theories – the unifying “high” theories in physics – to defend the view that a unifying theory may be as good at this job as can be and yet not be true. It can give us an excellent picture of the world as a whole, so long as we do not then expect to see the details correctly. Cartwright based her claims on the way she saw scientific modeling working in practice. The behavior of the planets does not ‘follow from’ the laws of mechanics. Rather, *we* derive the details of their behaviour. We do so starting from those laws, but in the course of our derivations we distort what the laws say. The corrections are not unmotivated. A great deal of knowledge from other domains, and lots of experienced practice, goes into it. But they are ad hoc from the point of view of mechanics.

Even though, on an account like Cartwright’s, the laws are not true, still they may be the very best and indeed an excellent – and thus ‘the correct’ – way to see ‘as one’ all the disparate phenomena we derive from them. We might liken this to the kind of realism about the choice of laws that many advocates of the Mill-Ramsey-Lewis ‘best system’ account of laws seem to adopt. On the Mill-Ramsey-Lewis account, the laws are the simplest set of claims from which we can derive the widest set of phenomena. Of course one may suspect that there is no ‘best’ system. But many act as if there is and that fundamental physics is on its way to finding it, perhaps even to finding *one* ‘simple’ system from which all facts can be derived. What we’d like to point out is that this kind of realism about the system – that there is one unique best one – is independent of whether the lower level facts ‘follow from’ the unifying laws, as Friedman pictures it, or we derive them, with distortions, as Cartwright sees it.

So, a theory may give us an understanding of a domain ‘as whole’ without being true to any of the phenomena in that domain. This can be classed a kind of realist understanding, supposing that we can be right or wrong about what the best picture of the whole is. How does empirical adequacy fare for this kind of understanding? The answer is immediate. The unifying theory that provides the understanding will generally be far less empirically adequate than the lower-level theories it unifies.

Perhaps we should recall at this stage that we are not committed one way or the other to realism about any of the items we discuss. For those who think that there is no right or wrong about the choice of a unifying theory when no such theory is true, it seems the understanding provided by unification would then be, in our system of classification, not ‘realist’ but rather ‘pragmatic’: it make things more comprehensible *to us*. But in this sense it seems to need no argument that a theory can at one and the same time improve comprehensibility and diminish empirical adequacy.

##### The natural classifications for laws

This is the central job of theory according to Pierre Duhem, who thought that successful physics categorizes empirical laws in a way that progressively reflects an underlying ‘natural’ classification and it is still reflected in how physics theory is organized today. Consider for example theories in physics with well-known names: Newton’s theory of gravity, Maxwell’s theory of electromagnetism, Einstein’s theory of relativity, quantum gravity, or string theory. Whether these theories are true or not, they organize empirical phenomena under them in a way that allows for subject-specialization in physics and the detailed comprehension that goes with it that promotes new visions, new practices, and what gets called ‘the growth of knowledge. Even if one argues that this is a realist understanding – that there is a *right* way to sort laws together into separate categories (as some take to be Duhem’s view^8^) – as with the overall picture, when we formulate laws in ways that make them fit in tidy categories, the laws so formulated may be less empirically adequate than when they could be formulated just so, so as to get the empirical phenomena exactly right.

##### Patterns

It is sometimes said that theory supplies understanding by revealing the patterns in nature. On this view, in Philip Kitcher’s (1989) words, “Understanding the phenomena is not simply a matter of reducing the "fundamental incomprehensibilities" but of seeing connections, common patterns, in what initially appeared to be different situations.” (pp. 81–82) Often these patterns are taken to be real: there is a fact of the matter about what patterns there are and just what they are like.^9^ In this case the understanding supplied is a realist understanding.

Still, when a theory supplies understanding by correctly showing patterns in the world, just as when it supplies understanding by reducing the number of independent variables or showing the overall picture or getting the laws placed in the right categories, it may well be less empirically adequate than a theory that aims just for empirical adequacy with no attention to making the patterns visible. The reason can be similar in all these cases. As with Elgin’s “felicitous falsehoods”, often the best way to bring out similarities and differences or to show overall patterns or how things fit together is by using a representation that is an average, or blur, or idealization of the real things that is not true to any of them. This is widely recognized in the case of seeing the trees as a forest, where it is clear that in seeing them as a forest we both lose and misrepresent a lot of empirical detail. Similarly, we can see and appreciate the pattern even if each individual piece is not entirely accurately represented and departs in various ways from it. And seeing things together that are very much alike is a way of understanding them that is in no way dependent on either the truth or the empirical adequacy of the vehicle that unites them.

### Counterfactual understanding

Often there is understanding of the world to be had from vehicles *not owing to* their having any proximity to truth or empirical adequacy – so the vehicles need not be remotely true or empirically adequate. One kind of understanding that fits this bill is *counterfactual understanding*: understanding that comes from being able to see counterfactual possibilities. (See Lipton (2009) for a fairly detailed account of this kind of understanding.) We will consider counterfactual understanding of three different kinds.

#### Understanding via vehicles that provide simple make-believe models

One way to provide counterfactual understanding is by constructing simple make-believe, often very diagrammatic, worlds, frequently described in highly abstract terms or, where more concrete terminology is used, the descriptions are meant to carry little of their ordinary content.

Consider Akerlof’s (1970) model of the car market. The model pictures an abstractly described cause – asymmetric information – and a concretely but thinly characterized effect: a big difference in price between new and slightly used cars. In the model, asymmetric information in constituted by the seller of the car having much relevant information about its condition; the buyer, little. As a result, the price a rational buyer will offer for a used car depends on the average quality of used cars on the market; the price that a seller will accept depends on the quality of that particular vehicle. Therefore, one will not sell a used car that has a quality higher than the average across the used car population. Rational buyers, knowing this, will further reduce the price they offer, which causes sellers to withhold even more cars and so on. Ultimately the market collapses and no used cars are offered for sale.

This is of course a bad prediction about the sale of used cars in the real world. In so far as we think that the difference in knowledge about cars is working in real world cases as it does in the model but results are different because the model ignores the other causes at work (e.g., used car salespersons’ care for their reputation) we could place this example in the category of partial realist understanding. There are a few reasons for putting it here instead. First is that it does give us counterfactual understanding of whether and how asymmetric information affects car sales in a world where no other causes are present. The idea is that *if* we lived in a world where asymmetric information were the only cause, then the used car market *would* collapse. This gives us not just (realist) understanding of this alternate world, but also (counterfactual) understanding of *our* world. For instance, with this model in view a government might successfully implement very strong full disclosure legislation for car sellers that eliminates asymmetric information. The Akerlof model would then give us very good counterfactual understanding of why that car market works so well, even though it does not depict any mechanism that exists there – this particular market works well in part because the government has eliminated the opportunity for sellers to have better information about their cars than buyers do. Striving for empirical adequacy would hinder the model from illustrating this possibility.

A second reason is that the model is not used primarily to understand car markets but rather to understand what happens, or could happen, or does not happen in a huge variety of quite different situations from asset pricing to the signing of the Magna Carta. This contrasts with the usual examples of Galilean idealization; for instance, the model of how bodies *move* when gravity alone is at work is generally used to help us understand real world *motions*. But Akerlof’s model about what would happen to a car market were differences in knowledge of cars’ features and history at work unimpeded is supposed to help us understand why the Magna Carta was signed.

Independent of where the understanding these models provide should be catalogued, it should be clear that models like this will not get better at supplying that kind of understanding just by increasing their empirical adequacy, and for the most part, they would probably get worse at it.

Another example of a model that can be seen as giving counterfactual understanding is economist Thomas Schelling’s (1978) checkerboard model, which gives a story about racial segregation. It is easy to see how neighborhoods would be racially segregated if individuals have strong discriminatory preferences. But this simple scenario does not make us understand how people could be segregated if they prefer mixed neighborhoods. To understand this, Schelling distributes nickels and dimes on a checkerboard. Coins are moved to new locations where they are less outnumbered depending on how many of their neighbors are the same denomination. Schelling found that even when coins are not moved unless 2/3 or more of their neighbors are different, ultimately the coins bunch into neighborhoods all of the same denomination.

This model talks about coins on a checkerboard and predicts how their locations will bunch. It makes no empirical predictions about people and segregation. What it does is far more subtle and interesting. It deals with a possible situation. As Aydinonat Emrah (2007) notes, the model is constructed to provide insight into how certain individual mechanisms that real people *may* display (i.e. individual tendencies to avoid a minority status) *may* interact under certain conditions to produce segregation. But that’s not what the descriptions in the model stand for.

Do we slip into relativism here? Can any old model that constructs a make-believe world give us counterfactual understanding of some aspects of the real world? No: Schelling’s model makes a *plausible* conjecture that segregation may be the unintended consequence of even mild discriminatory preferences – and this is based on our familiarity with *real* people and their preferences. In this way it is consistent with our real world in key respects, like its assumptions about people.

The Schelling model does not tell us that mild discriminatory preferences *do* result in large segregation, but it opens our minds to a previously unimagined possibility – who’d have thought that such mild racial preferences *could* lead to complete segregation in a world quite similar to ours? Here then is a case of a celebrated model in economics – celebrated for providing insight into, and what we are labeling ‘counterfactual understanding’ of, racial segregation – where empirical adequacy simply does not figure. The model is empirically sterile with respect to the issues it gives insight into – making no predictions about them at all.

#### Understanding via vehicles that show up impossibilities revealed by ‘failed’ ideas from the past

Consider again the Rutherford model. In addition to the realist understanding it gives, it also gives us counterfactual understanding. Owing to its empirically incorrect prediction about the inward spiraling of the electron, it shows how an atom *couldn’t* be: it illustrates a physical impossibility. While the model was proposed by Rutherford in the hopes of giving *realist* understanding understanding since it, today we can use it for gaining counterfactual understanding: *if* electrons revolved around the nucleus the way the model says they do, then matter *couldn’t* exist as we know it.

We’d again like to stress that this plays a particularly significant role in science teaching. The Rutherford model was an important step in the evolution of ideas about atomic structure in the history of science, as it is in a science learner’s progression of ideas about the phenomenon. In Kuhnian jargon, within the Rutherford paradigm – given the results of his gold foil experiment – the model seemed very plausible. So before being introduced to the idea of an accelerated charged particle losing energy, students of science will likely appreciate Rutherford’s model. Once they are introduced to this conflicting idea of the spiraling electron, they will be able to see why an atom simply couldn’t be the way Rutherford thought it was. Similar arguments can be made about gaining understanding from other ‘failed’ ideas such as luminiferous ether, the heliocentric model of the solar system, and so on. The understanding we gain here seems especially significant when we look at the study of science as a study of the evolution of scientific ideas. And it’s worth noting that understanding here squarely *depends* on empirical inadequacy!

#### Understanding via vehicles that provide plausible explanatory stories

Empirically inadequate plausible explanatory stories can give us understanding by showing how things can be consistent with facts we insist on, such as accepted theory. Consider the MIT bag model. It describes hadrons (particles like protons and neutrons) as ‘bags’ in which (two or three) quarks are spatially confined, forced by external pressure. This takes into consideration the fact that quarks have never been found in isolation and are hence thought to be spatially confined. With the help of boundary conditions and suitable approximations, the single model parameter (bag pressure) can be adjusted to fit hadronic observables (e.g. mass and charge). Stephen Hartmann (1999, 336) observes that the predictions of the model only very modestly agree with empirical data. By normal empirical standards, the model fares badly. Are quarks really confined the way described in the model? We don’t know – and if empirical adequacy is a guide to truth, then very probably not.

Hartmann asks why physicists entertain the model, despite its empirical shortcomings. His answer is that it provides a “plausible story” by which it enhances our understanding. The bag model is a “narrative told around the formalism of the theory”; it is consistent with the theory; and importantly, it gives a plausible, intuitive, and visualizable picture of a hadron as quarks confined in a bag. Here we also get modal understanding. To the question, ‘How could quarks be spatially confined?’, this model answers, ‘Possibly, as if they were in a bag’. The answer is a good one because it is easily visualizable and because it illustrates a possibility about quark confinement.

A common response is that the model is itself understandable, but it does not provide understanding of the target if it is not reasonably empirically adequate to it – we understand the model, but not the target. No, Hartmann contends: “[A] qualitative story, which establishes an explanatory link between the fundamental theory and a model, plays an important role in model acceptance” (1999, 15). That’s because the model gives a story that relates to the known mechanisms of quantum chromodynamics, the theory that is fundamental of this domain.^10^ Not any old model that’s visualizable and intuitively plausible will do the job: although empirically inadequate, the bag model is consistent with many things the theory says. It may be too bad for the realists and empiricists that this model is not empirically adequate, but theoretically, there is little reason why the quarks *couldn’t* be confined this way. Here again is a model highlighting a theoretically as well intuitively plausible possibility.

### Pragmatic understanding

The final kind of understanding in our catalogue, described by de Regt (2014), comes from using a theory or model for practical use and manipulation, which lines up closely with the other aim of science we discuss in this paper – managing the world. There is understanding to be had of the world via a vehicle that helps us manipulate and control it – call this *pragmatic understanding*. De Regt associates understanding with the intelligibility of a theory. Intelligibility in this case is pragmatic and contextual. It consists in knowing how to use the theory for prediction and manipulation/control – so understanding for him is a skill.

As mentioned earlier, de Regt advances arguments similar to Elgin’s: he criticizes and rejects the realist thesis regarding understanding. He then shows how, in trying to predict and control (parts of) the world, we employ models and theories that are judged to be false. “Whether or not theories or models can be used for understanding phenomena does not depend on whether they are accurate representations of a reality underlying the phenomena,” (2014, 16) he maintains. (He gives many examples of false theories used for domain-specific manipulation and prediction, like Newtonian mechanics.) *Nor, we add, does it depend on whether they are empirically adequate.* Although de Regt doesn’t explicitly say much about the empirical adequacy of theories and models for understanding, the arguments he gives against requiring a vehicle of understanding to be true apply to requiring it to be empirically adequate as well. We shall say no more about this here because we continue this line about models and theories for use and manipulation in the next section.

Unification can also supply a kind of pragmatic understanding. As Mary Morgan (2010) points out in her work on the travel of facts and techniques from one domain to another, it can be extremely useful to see that the laws grouped together under the same unifying claim are similar in significant ways. It allows us to use similar methods of study, modeling strategies, approximation techniques, and the like, and it suggests analogous predictions to look for from one domain to another. This suggests new concepts, new theories, and new methods; it helps us advance our sciences at both a theoretical and a practical level.

Note that, just as with realist unificatory understanding, a unifying theory can supply pragmatic understanding while diminishing empirical adequacy. When it comes to borrowing techniques, looking for predictions in one domain analogous to those already established in another, and the like, it is the analogies among the unified sub-theories that matter, not the empirical adequacy of the unifying theory. The unification may be substantially less empirically adequate than the ones being unified.

## Managing the world around us

Yes, we advocate understanding. But ‘the’ task of science is not to understand the world any more than it is to represent the world as it is. There are a great number of tasks we can, and should, ask science to undertake. Many can be grouped under the broad aim, ‘to change the world’. As mentioned above, according to de Regt (2014) the ability to manipulate and control also gives us (pragmatic) understanding of the world– but we take this to be a big goal of science in itself, and one we especially care about.

To advocate managing the world as a task for science is to advocate a kind of instrumentalism, not in the traditional sense, as an epistemological doctrine, but in the sense of Julian Reiss, who teaches, “[S]cience strives ‘to build a ‘toolbox’: its theories, models, statements or results aim to provide its users with devices for orienting themselves in this world and to mold it into shape according to their values and aspirations.” (2012, 364)

Do we need theories that make true theoretical claims in order to mold the world like this? No. Here we rehearse the outlines of arguments well known from the works of pragmatists and instrumentalists. To manage the world what we need are knowledge and practices that we can rely on to generate models that make correct enough predictions about what will happen when we act. Sometimes we are reassured that a model will make correct predictions because we have good reason to think that the facts supposed in its construction are true. But notoriously, models using radically false assumptions about the world can make accurate predictions about targeted outcomes.^11^

This is richly illustrated by Gigerenzer et al. (1999) in his work on cheap heuristics that make us smart. It is also famously argued by Nobel prize winning Chicago School economist Milton Friedman (1953) about models in economics. Leaves may not chase the sun. But assuming that they do so, he argues, is a good predictor of where they will be facing. Similarly, he maintains, it may not be true that people making economic decisions are ideally rational. But assuming them to be so can give very good predictions about various specific economic outcomes, even though, as with Prescott’s business cycle theory, it gets a good many other empirical predictions dramatically wrong. More recently in economics, Oxford econometrician David Hendry (2002) urges that it is best for forecasting in certain domains of economics to adopt false models that have many false empirical consequences rather than to try to model the true causes. That’s because in these domains the causes change often, quickly, and unpredictably whereas the kinds of far less accurate forecasting models he advocates ‘catch up quickly’ to allow reasonably accurate forecasts of the target features. His models, he claims, are good enough for what we want to do but they don’t even attempt a true picture of what’s going on and they give very false predictions about many non-targeted features.

So, to manage the world we don’t need our theories to get right most of their empirical predictions. We need them to be right about exactly the empirical facts we rely on in order to bring about the results we want. This may mean that we need to get some theoretical facts right as well. But even in the cases where some theoretical truth is needed, at most this involves getting right specific theoretical facts that imply those conclusions we need to do the job at hand. This is not to say that empirically adequate theories are of no use in managing the world. After all, if a theory gets all the empirical facts right, it is bound to get right the ones you need on this occasion. On the other hand, looking for a theory that gets them all right rather than ones that reliably provide the predictions you need is expensive overkill, as in our example of buying a Swiss army knife to cut bread.

This underlines one of the reasons we find Douglas’s attitude odd by her own standards. She urges that a theory that is not empirically adequate “must be corrected to meet the minimum requirements as quickly as possible. This doesn’t seem to fit with her own views about the importance of values in science, especially the importance of considerations about what we value, and how much we value it, in setting standards for theory acceptability. She is well known for arguing that standards of acceptability should depend on the uses to which a theory will be put (2006; 2009). In particular, she is concerned with the question, what is the cost if a wrong theory is used versus the cost if a theory is rejected yet is correct. For Douglas these costs must be taken into account in deciding whether to use a theory or not. Yet it seems for her they do not play a significant role in deciding whether to make do with a theory that solves the problems we face rather than carry on the hunt for one that provides extras that we do not need.

There are of course the usual rejoinders: we never know when a piece of knowledge may come in handy; we do – and should – value knowledge for its own sake; sometimes the fact that we have no use for at the moment is just the clue we need to come up with newer, better theories; or it may be the missing piece that we need for understanding. Yes all that is true. And were there no costs in time, effort, money, and talent to hunting theories that go beyond our instrumental needs, perhaps we should for just these kinds of reasons indulge in empirical adequacy. But there are costs, and the same kinds of reasons that move Douglas to count the costs in deciding on theory acceptability argue that we should count the costs before pursuing empirical adequacy.

There is another epistemic version of the argument for overkill, this one analogous to the case of the shopper who values above all else not having to judge which shops to trust. If you knew that your theory was empirically adequate, that would answer big questions for you: which of its empirical consequences can be trusted for use? They all could. That of course is a big IF. And it’s a costly way to decide that a prediction can be trusted: invest in the hunt for an empirically adequate theory and in all the empirical and theoretical work it takes to warrant confidence that it is so. It is generally cheaper to follow familiar practices: trust the consequences of the theory in situations where we have a great deal of experience that they give good enough results, build prototypes, overbuild -- put safety nets in place -- in case we are wrong, proceed with caution, and pray. We urge that this is the safer policy over slavish trust in the putative consequences of well-confirmed theory. Also that it is the morally correct one where results really matter. When people’s lives and welfare are at stake, we should check, check, and double check.

Finally, we should rehearse an old theme from Otto Neurath. It is theories that are empirically adequate or not. A theory is empirically adequate if it gets right a good enough number of the central empirical facts it predicts. But facts we need to know to mold and manage the world are seldom in the domain of any one theory. Even if you have marvelous theories you can’t just derive what will happen in most real situations from them. The theories may provide useful tools but you need to build the prediction, not derive it. This will generally take knowledge from a variety of different theories, local and concrete knowledge, skill, experienced practices, luck, finesse, and, as above, a great deal of trial and error, check, and double check. So even if we follow Douglas’s injunction to correct our theories as quickly as possible to get them up to the standard of empirical adequacy, this will generally not meet the instrumentalists” needs.

Returning to the realist-antirealist divide mentioned at the start, our argument here distances us from the realist and antirealist alike. When it is action that we are interested in, all and only facts that impact action become important. When it comes to the aims of science, we are realist about both theoretical and empirical facts. If we need to get facts about some unobservable microbes right in order to cure a stomach ache, then we had better strive to get them right. We are equally anti-realists about both empirical facts and theoretical facts: those we don’t need. To manage the world, our theories have no need to be either true or empirically adequate. Again, as with the task of understanding, there is no privileged place for empirical adequacy in theory choice when we are trying to change the world.

## Some parting thoughts

One might be tempted to think that empirical adequacy can be salvaged as a sine qua non of theory choice by adopting a more relaxed characterization of empirical adequacy.^12^ We think this won’t work for a couple of reasons. First, as we argued, some jobs that scientific models or theories are expected to do don’t require them to get any empirical facts right, like Galilean thought experiments, which necessarily distort the setting in which the effect occurs so the effect predicted is different, often dramatically, from the effect that happens. Second, we repeat that we do not deny that often, maybe always, a model or theory has to get some facts right to be acceptable. But again, what kinds of facts these are will vary with the purposes to be served. There is a relaxed formulation of an adequacy criterion that works, but it is trivial and it does not focus on the empirical: “For a theory to be acceptable, it must be right (enough) about those facts that it needs to get right to do the job in view.”

To make our point clearer, it might help to reframe our opening question “What's so special about empirical adequacy?” as: “What's so special about the empirical consequences of a theory?” The answer presumably is that the empirical is our epistemic access to what the world is like; the empirical consequences of a theory/model are our indirect check that things are as it says. But for many jobs, we do not require this kind of indirect check that what the theory/model says about the facts we need for the job is likely to be right. We can have many other reasons for expecting this (like frequent success in the past or a good enough grasp on what’s happening to see why the model should get right what we need). Of course the empirical consequences of a model matter to managing the world with it. But as we have stressed, not all of them. A theory/model that gets right whatever it is we need to get right may get most if not all of its empirical consequences wrong, even its most central ones.

As we have stressed, there are many jobs that science is called on to perform, and which is most important at which place and time depends on context. Yet little attention is paid in the philosophy of science to what makes a theory fit for these jobs. True, we do sometimes discuss “virtues” that may be desired in a theory beyond empirical adequacy, but this is almost always in the context of the underdetermination issue: how to choose among theories that are empirically adequate.^13^ We find this odd. One may argue that the virtues called into play in these cases are sufficiently truth indicative that possessing them makes it likely that the claims are true. But that is a hard argument to make and failing that argument, it seems we knowingly pick from among a great many incompatible theories – including all the unconceived ones – that seem to have equal claims to truth, one theory that has some extra virtue like simplicity or plausibility. That is what’s odd. If in the end we are going to choose one theory from a bunch of others that have equal claims to truth because it does some job we want to do, why constrain the choice to just the theoretically true or the empirically adequate ones to begin with? Why not go for the best we can conceive that will do the job at hand well, regardless of its truthfulness about either theoretical or empirical facts unnecessary to the job?

## Conclusion

We suggest two take-home messages:

- Truth is no trump, nor is empirical adequacy. These form one part of what we aim for in doing science and in investing in it. For much else, empirical adequacy has no special role to play and alternatives are very welcome if they can do the job. So what’s so special about empirical adequacy?
- We need to keep hunting for better theories and keep constructing better models. But in doing so, we must not put all our eggs in the empirical adequacy basket. There’s no sufficient reason to think that”s the best path to models and theories that will help us do what we want.

## Footnotes

1 Van Frassen (1980) definition – a theory is empirically adequate if it gets right the claims the theory makes about observable objects – is often referred to. But we think that this is not really what is generally meant since it demands getting right all these claims, including the infinite host of claims about never-to-be-observed observables; and also because many of the facts theories imply about observable objects involve properties almost anyone would label as theoretical.

2 See for instance Khalifa (2013), Grimm (2010), Strevens (2013)

3 De Regt (2014) refers to (a more general version of) this as ‘the realist thesis regarding understanding’ and has argued against it at length, specifically focusing on what we call ‘pragmatic understanding’, which we get to later in this paper.

4 Though they could both provide partial, but only partial, realist understanding and still be empirically adequate if they introduce the right compensating ‘falsehoods’ to correct the predictions without obscuring the features that provide the partial understanding.

5 Thanks to an anonymous referee for this formulation.

6 Also long argued by Cartwright (1983), Morrison and Morgan (1999), Wimsatt (2007) and others.

7 Also see Cartwright (1983) for similar arguments

8 Though Bhakthavatsalam (2015) argues against this simple reading of Duhem.

9 See for instance, Ladyman et al. (2007), Ishida (2007).

10 See Hartmann (1999) p. 10 for a detailed discussion of this.

11 See Robert Northcott (2016) for defense of similar claims to ours.

12 Some people including an anonymous referee for this paper have suggested this to us explicitly.

13 See Matthew Brown (2012) for a critique of such a setting.
